# A naturally-occurring mutation in *Cacna1f* in a rat model of congenital stationary night blindness

**Published:** 2008-01-09

**Authors:** Yonghao Gu, Lifeng Wang, Jie Zhou, Qun Guo, Na Liu, Zhenqiang Ding, Li Li, Xinping Liu, Jing An, Guolin Yan, Libo Yao, Zuoming Zhang

**Affiliations:** 1 Department of Clinical Aerospace Medicine, Fourth Military Medical University, Xi’an, China; 2 Biochemistry and Molecular Biology, Fourth Military Medical University, Xi'an, China; 3 Department of Ophthalmology, Anhui Provincial Hospital Affiliated to Anhui Medical University, Hefei, China

## Abstract

**Purpose:**

To identify the gene mutation responsible for a previously described rat model of X-linked congenital stationary night blindness (CSNB).

**Methods:**

Rat orthologous genes for *Nyx* and *Cacna1f* were isolated from retina through rapid amplification the cDNA ends (RACE) and examined for mutations. Electroretinograms were used to identify affected animals.

**Results:**

The rat *Nyx* cDNA spans 1,971 nucleotides and encodes a protein of 476 amino acids (GenBank: DQ393414). The rat *Cacna1f* cDNA spans 6,076 nucleotides and encodes a protein of 1,980 amino acids (GenBank: DQ393415). A c.2941C>T (p.R981Stop) mutation in *Cacna1f* was found in affected rats. Immunochemistry study showed labeling for rod bipolar and horizontal cells were reduced in affect retinas. For affected rats, b-wave and oscillatory potentials of scotopic ERG were absent, and b-wave of photopic ERG was clear but obviously reduced.

**Conclusions:**

The *Cacna1f* mutation identified in the rat model of CSNB was predicted to lead to a protein product that is shortened by 999 amino acids, indicating that this is a model for the incomplete subtype of human X-linked CSNB (CSNB2). This rat model will be useful for defining the pathophysiological properties of this human disorder.

## Introduction

Congenital stationary night blindness (CSNB) encompasses a group of inherited, nonprogressive retinal disorders that primarily affect night vision [1] and can be transmitted in autosomal recessive, autosomal dominant or X-linked modes [2-7]. The X-linked form of CSNB is frequently associated with myopia, nystagmus, decreased visual acuity, and occasionally strabismus [8-10]. Based on functional and clinical information, Miyake et al. [11] divided X-linked CSNB into two types: complete (CSNB1) and incomplete (CSNB2). CSNB1 is characterized by normal to mildly subnormal cone function and the complete absence of rod function. It is caused by mutations in the *NYX* gene, encoding a glycosylphosphatidylinositol (GPI)-anchored extracellular protein [12,13]. CSNB2 patients retain measurable rod function with significant impairment of cone function, yet have mutations in the *Cacna1f* gene, which encodes the α_1F_ subunit of an L-type calcium channel [14,15]. Recently, mutations in GRM6, coding for the metabotropic glutamate receptor mGluR6 [16,17], and CABP4, encoding a calcium binding protein [18], have been identified as the cause of autosomal recessive CSNB (arCSNB) leading to phenotypes similar to CSNB1 and CSNB2, respectively.

We recently reported a naturally occurring rat model of X-linked CSNB [19]. This model was originally identified by electroretinogram (ERG) recordings obtained from a single outbred Sprague Dawley rat, and the trait has since been inbred for more than 16 generations. The ERGs obtained from the original mutant showed a marked loss of the rod b-wave with relatively normal cone ERGs, and were interpreted to resemble most closely the human CSNB1 phenotype. During the inbreeding process, however, it became clear that the cone response of mutant rats was also compromised such that the overall phenotype more closely resembled the CSNB2 phenotype. In the present study, we isolated the rat orthologous genes for both *Nyx* and *Cacna1f* and examined these for mutations in affected rats. As will be described, our results indicate that this rat model of CSNB is caused by a *Cacna1f* mutation.

## Methods

### Animals

Affected and control rats were obtained from the 14^th^ inbred generation derived from the originally identified mutant male [19]. Since the defect is inherited as an X-linked trait, the mutant line has been maintained by mating affected males to control females and then mating carrier females to affected males. All procedures involving the animals were approved by Animal Care and Use Committee of the Fourth Military Medical University and were in accordance with the ARVO Statement for the Use of Animals in Ophthalmic and Vision Research.

### Electroretinography

ERG recordings were used to study the phenotype of ten affected and ten control male rats, that were 10 weeks of age. ERGs were recorded using procedures described previously [20]. After 10 h dark adaptation, rats were anesthetized intraperitoneally with ketamine (70 mg/kg, Sigma, Saint Louis, MO) and xylazine (10 mg/kg, Sigma). The pupils were dilated with 0.5% tropicamide, and animals were secured to a platform with a heating pad to maintain the body temperature. ERGs were recorded from the corneal surface using a silver-chloride electrode loop that made contact through a layer of 1% methylcellulose. Stainless steel needle electrodes placed in the cheek served as reference leads while those placed in the tail acted as ground leads. ERGs were recorded by a commercial system (RETIport; Roland Consult GmbH, Brandenburg, Germany) using a band pass of 0.5 to 1000 Hz. Strobe stimulus flashes were delivered in a Ganzfeld, and neutral density filters were used to control stimulus intensity.

A dark-adapted intensity series was recorded first, using a stimulus range of −2.5 to 0.5 log cd s mm^−2^. Interstimulus intervals increased from 15 s at the lower flash intensities, to 2 min at the highest flash levels. A steady adapting field (1.3 log cd mm^−2^) was then presented within the Ganzfeld. After a 10-minute period of light adaptation, cone ERGs were elicited by flash stimuli superimposed against the adapting field. Cone ERGs were recorded in response to stimuli ranging from −2.5 to 0.5 log cd s mm^−2^. In each case, the responses to 25 consecutive flashes presented at 2.1 Hz were averaged.

### Isolation of the *rattus norvegicus Nyx* and *Cacna1f* full-length cDNA

Smart Race technology (Clontech, Mountain View, CA) was used to amplify the full-length cDNA of *Nyx* and *Cacna1f* from the rat. Total RNAs were isolated from retinas of affected and control rats with Trizol reagent (Invitrogen, Frederick, MD) according to manufacturer's protocol. Primers were designed from predicted rat *Nyx* sequence (GenBank: NM_001100967) and available *Cacna1f* sequence (GenBank: NM_053701) in Table 1. The PCR products were subcloned into the pMD-18T vector (Takara, Dalian, China) and transformed into *E.coli*, and sequenced.

**Table 1:** Primers used to isolate the rattus norvegicus *Nyx* and *Cacna1f* full-length cDNA.

**Gene**	**Purpose**	**Primer (5′-3′)**	**Location**
Nyx According to (NM_001100967)	5′ RACE 3′ RACE For middle region	F: AGGAGACGCTCGGGCACGCTGAAG	(nt496 to nt159)
		R: GCACCCTCAATCTGGGCGGCAAC	(nt825 to nt847)
		F: CCACAACAACCTGTCCTTTATTAC	(nt337 to nt360)
		R: TCAGTCCCTCTGTGGACCCAAC	(nt1480 to nt1501)

Cacna1f According to (NM_053701)	5′ RACE 3′ RACE For middle region	F: TGGCTTCCACTCCACAATGCTGATG	(nt284 to nt308)
		R: CAGTGACCTGCTGGCACAGAGAACC	(nt5861 to nt5885)
		F: AGAGGATGTCGGAATCTGAAGTCG	(nt25 to nt48)
		R: GGACCTTCGGGGCTACCTGGAC	(nt1226 to nt1247)
		F: TGGAAGCGGATAAAGCAGAAAT	(nt4698 to nt4719)
		R: TCAAAACTGTGAACTGGACAAGAA	(nt2470 to nt2493)
		F: TGGAAGCGGATAAAGCAGAAAT	(nt4698 to nt4719)
		R: GGGGGTGTCTGTTATGGAACCA	(nt1454 to nt1475)
		F: CCTTGCGAAGCGGGTTGGTTTG	(nt2592 to nt2613)
		R: GTGTTGAGGAGGATGAGCAGAA	(nt3616 to nt3637)
		F: TGGATGAGAAATGTGGCATAGAA	(nt4773 to nt4775)
		R:CATCAAAGCGGGAGAGAATAGACT	(nt5908 to nt5931)

Full length cDNA of rat *Nyx* and *Cacna1f* were isolated by 5′ and 3′ rapid amplification the cDNA ends. Primers were designed according to available sequences and manufacturer's protocol.

### Bioinformatics

The open reading frame (ORF) and translated amino acid sequences were predicted by the National Center for Biotechnology Information's (NCBI) ORF Finder and DNAStar 2.0 program. The nucleotide and amino acid sequences were aligned in NCBI's Blast program to search for sequence matches. The chromosomal location and extron-intron structure were analyzed in the NCBI's Genomic Biology. The sequences to be analyzed were retrieved from GenBank by NCBI's Entrez system. Multiple alignments were performed with Clustal X (1.8) program. Motif searches were carried out with ExPASy and Smart program.

### Mutation screening

To identify whether a mutation in *Nyx* or *Cacna1f* gene was present in the CSNB-like rat, we designed primers to amplify fragments encompassing the full-length cDNA from both control and affected rat (Table 2). The PCR products were subcloned into TA vectors (pMD-18T, Takara) and sequenced as described in the previous section.

**Table 2:** Primers used to amplify the full-length cDNA of rat *Nyx* and *Cacna1f*.

**Gene**	**Primer**	**Position**	**Sequence (5′-3′)**
Nyx	TN1F	1–26	GAGAAAGAAAAATAAGCAGTCAAACC
	TN1R	1032–1056	GATGCTGTTGCGATCTAGGTAAAGC
	TN2F	1219–1239	GAGTGGTTGCGTGATTGGATG
	TN1R	1946–1971	TTCTACTTTAATTTAGGCCTGTAGGC

Cacna1f	TC1F	1–25	GTGTGCAGATGGTCCTTCTATCTCC
	TC1R	1461–1480	CGGGGGTGTCTGTTATGGAA
	TC2F	1230–1249	GGACCTTCGGGGCTACCTGG
	TC2R	2366–2385	TTGGGGAGGGTTTCCTTCAC
	TC3F	2264–2287	GTGGCAACTACATCCTACTGAACG
	TC3R	3559–3579	CACGCGGTACTGATGTGGATT
	TC4F	3473–3497	ATCAAAACTGTGAACTGGACAAGAA
	TC4R	4249–4268	CCACAGGTAAATTCCTCGCC
	TC5F	4090–4111	CTTCAGGACGGCACACAGATAA
	TC5R	4777–1799	TGGATGAGAAATGTGGCATAGAA
	TC6F	4695–4717	AAAGATCTGGAAGCGGATAAAGC
	TC6R	5703–5723	CTGCCCCTCTTGCGGTGACTG
	TC7F	5468–5488	AACGCCAGGGCAGTTGTGAGG
	TC7R	5990–6014	GCAGGGAATTTATTGAGCGATAGGTA

Fragments of rat cDNA of *Nyx* and *Cacna1f* were amplified to cover the full length. Primers were designed according to sequences isolated in previous part.

### Histology

After 10-week old control and affected rats were killed by CO_2_ (5–10 min, in a sealed chamber), the eyes were enucleated, dissected along the ora serrata, and posterior eyecups were fixed in 4% paraformaldehyde at 4 °C for overnight. The eyecups were then rinsed in phosphate buffer (PB), dehydrated through a graded series of ethanol washes, and embedded in paraffin. A microtome was used to cut 5 μm thick sections, which were mounted onto slides and stained with hematoxylin and eosin for anatomy analysis.

### Immunochemistry

Sections were rehydrated, and 3% H_2_O_2_ in 40% methanol was used to block the endogenous peroxidase. After sections were incubated in blocking solution (10% goat serum, Boster) for 30 min, solutions were replaced and sections were incubated with primary antibody diluted in blocking solution at 4 °C for overnight. Primary antibodies used were a 1:5000 of anti-protein kinase Cα (PKC, Sigma) and a 1:3000 dilution of anti-calbindin D-28K (Chemicon, Southampton, UK). After washing with PB, sections were incubated in a 1:1000 dilution of biotin-conjugated secondary antibody (Boster, Wuhan, China) for 1 h. After a wash in PB, sections were incubated in an avidin-biotin peroxidase complex (Boster) for 30 min. Sections were rinsed in PB and immunoreaction was visualized with a diaminobenzidine-nickel solution (Boster) as the chromogen. The reaction in PB, before slides were coverslipped and images were captured digitally on light microscopy.

## Results

### Electrophysiology

**Figure 1:**
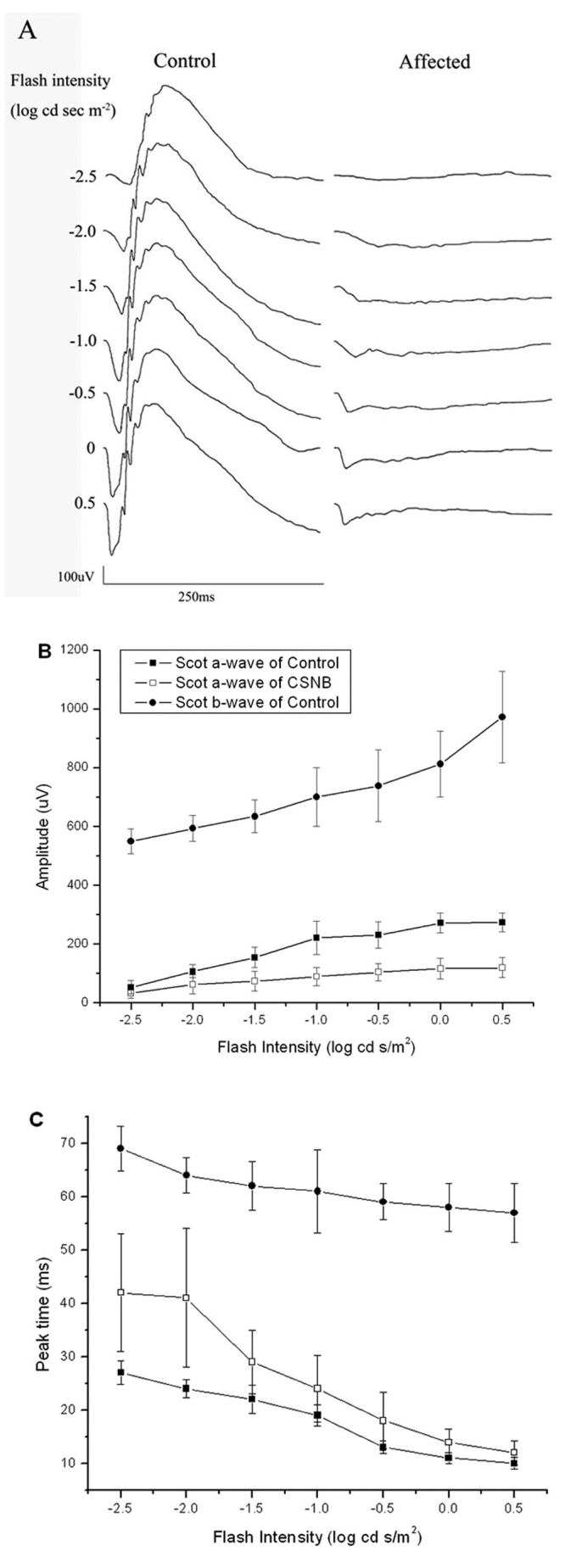
Dark-adapted electroretinograms. **A**: Comparison of dark-adapted ERGs recorded from control and affected rats. **B**: Amplitude of dark-adapted a-wave (square) and b-wave (circle) for control (filled) and affected rats (open). Data points indicate the mean±SEM response from ten 10-week-old rats. **C**: Peak time of dark-adapted a-wave (square) and b-wave (circle) for control (filled) and affected rats (open). Data points indicate the meanpom SEM response from ten 10-week-old rats.

Figure 1A presents ERGs recorded from representative control (left) and mutant (right) animals under dark-adapted conditions. Under dark-adapted conditions, ERGs of mutant rats lacked distinct b-waves or oscillatory potentials throughout the range of stimulus intensities. In response to high stimulus intensities, mutant rats generated a clear a-wave. In comparison to control responses, a-waves of mutant rats were significantly reduced in amplitude. Figure 1B,C show intensity-response functions for dark-adapted a- and b-waves of ten control and ten affected rats.

**Figure 2:**
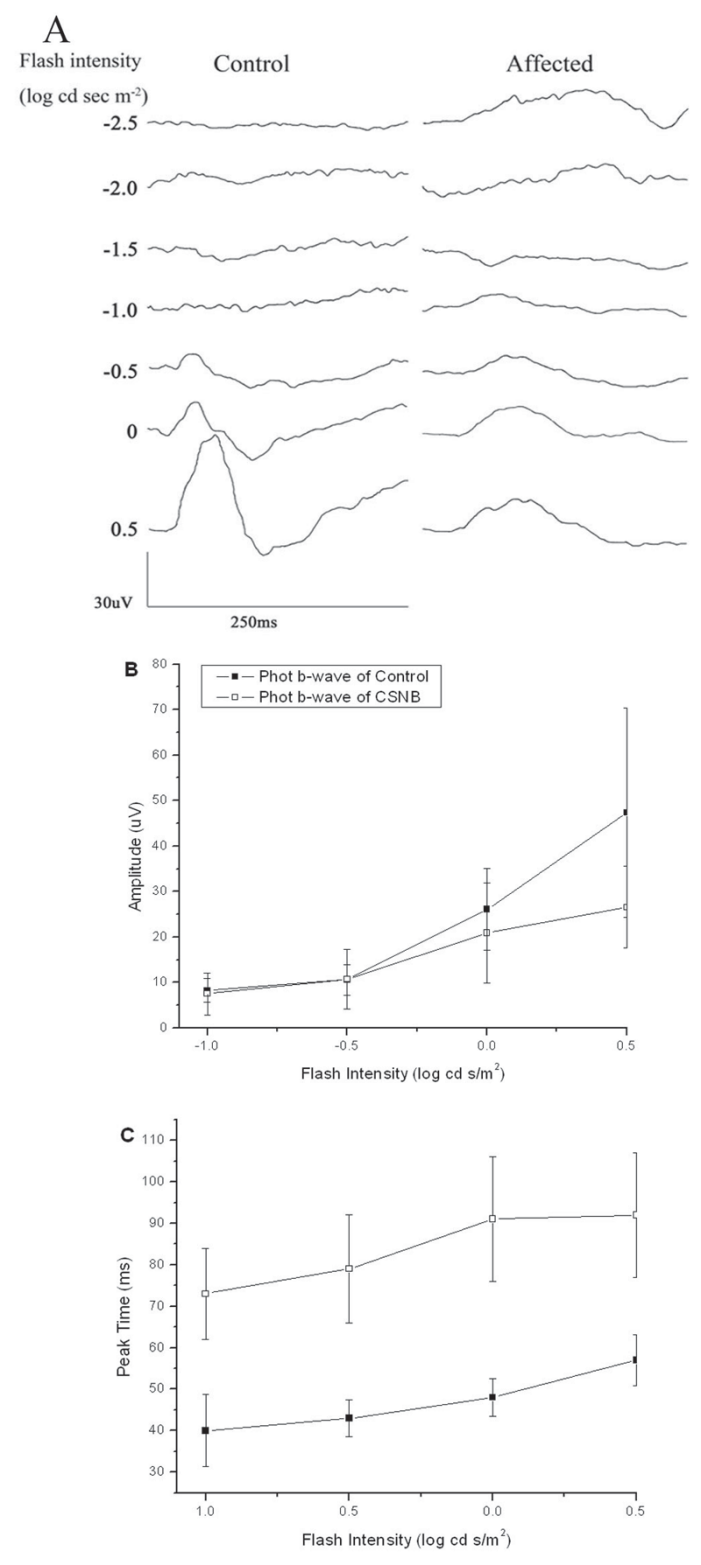
Light-adapted electroretinograms. **A**: Comparison of light-adapted ERGs recorded from control and affected rats. **B**: Amplitude of light-adapted b-wave for control (filled square) and affected rats (open square). Data points indicate the mean±SEM response from ten 10-week-old rats. **C**: Peak time of light-adapted b-wave for control (filled square) and affected rats (open square). Data points indicate the mean±SEM response from ten 10-week-old rats.

Figure 2A presents representative ERGs recorded under light-adapted conditions. In comparison to control responses, light-adapted ERGs of mutant rats were significantly smaller in amplitude and delayed in peak time. Figure 2B,C show intensity-response functions for the cone ERGs.

### Isolation of rat *Nyx* and *Cacna1f* cDNA

**Figure 3:**
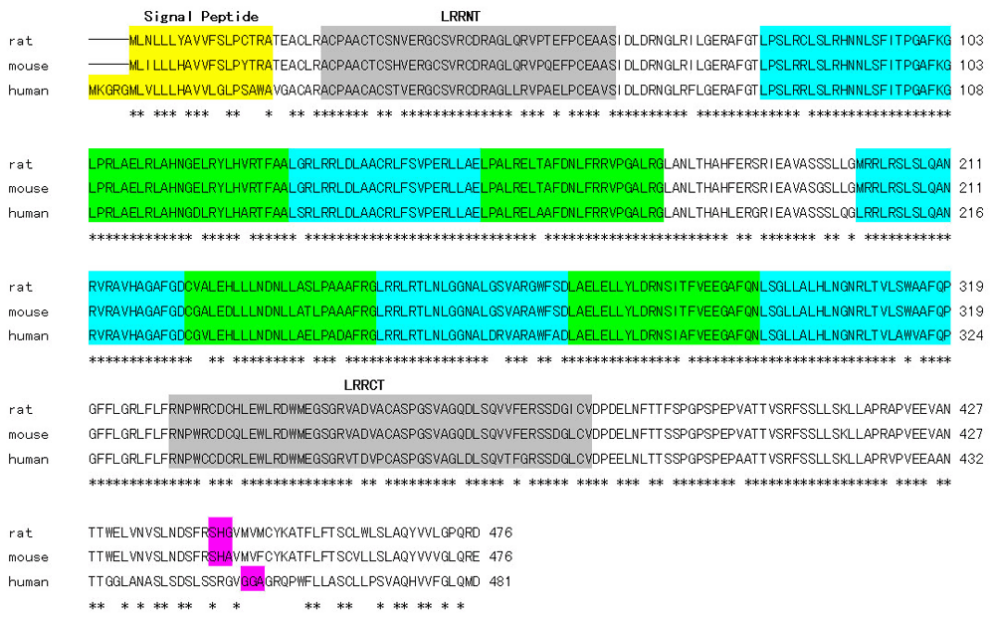
Alignment of rat, mouse (Genbank: NP_775591) and human (Genbank: NP_072089) predicted *Nyx* amino acid sequences. Conservative residues are marked by asterisks (*). Signal peptide is shown in white shading, N and C-terminal leucine-rich regions (LRRs) in gray, LRRs in green, predicted glucose phosphate isomerase (GPI) cleavage site in pink.

Full-length rat *Nyx* cDNA spans 1,971 nucleotides (GenBank: DQ393414). Compared with the computational predicted one (GenBank: NM_001100967), this sequence has complete 5′ and 3′ UTR. When aligned with the rat genome, it was predicted to locate in Xq12 and contain 3 exons. The ORF was confined to exon 2 and 3 as in human [12,13], mouse [21,22], chic [23], and zebrafish [24] genes, and encodes a protein of 476 amino acids with a predicted molecular weight of 52.5 kDa. The nucleotide sequence is 86% and 93% identical to human (GenBank: NM_022567) and mouse (GenBank: NM_173415) sequences, respectively. The translated amino acid sequence is 84% and 95% identical to human (GenBank: NP_072089) and mouse (GenBank: NP_775591) sequences, respectively (Figure 3). Computational protein motif analysis of rat *Nyx* predicted a characteristic domain structure: the N-terminal putative signal sequence, the core segment consists of 11 leucine-rich regions (LRRs) flanked by two cysteine-rich LRRs (LRRNT and LRRCT). Unlike human, the C-terminal GPI membrane anchor was not predicted in rat sequence. Only a potential cleavage site was found at C-terminal. Identity was much higher in the LRR core segment than in the signal sequence and C-terminal among the species. Recent work has suggested both human and mouse nyctalopin are membrane-bound extracellular proteins with function conserved [25], and orthologous nyctalopin proteins may have different mechanisms of cell membrane attachment [26].

**Figure 4:**
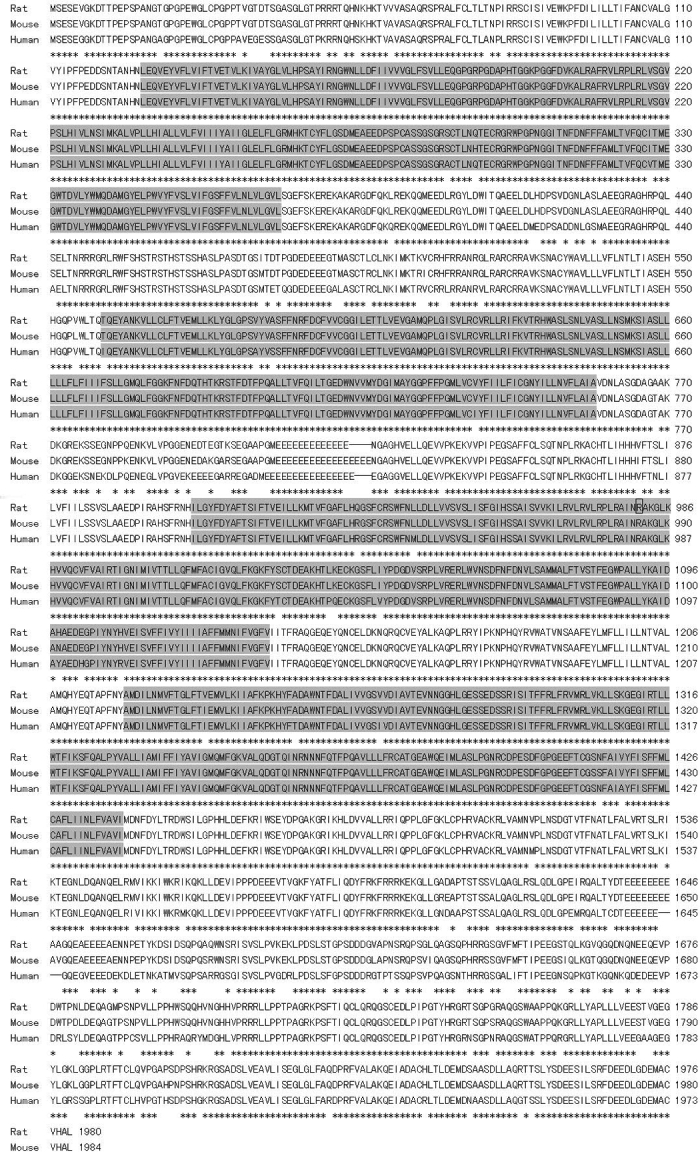
Alignment of rat, mouse (Genbank: NP_062528), and human (Genbank: NP_005174) predicted *Cacna1f* amino acid sequences. Conservative residues are indicated by asterisks (*). The putative transmembrane domains (Ion_trans) are shaded in gray. The box marks the position at which the normal protein is truncated in affected rat.

Full-length of rat *Cacna1f* cDNA spans 6,076 nucleotides (GenBank: DQ393415) and has complete 3′-UTR compared with available one (GenBank: NM_053701). When aligned with the rat genome, it was predicted to locate in Xq13 and contain 49 exons. The ORF was confined to all of the exons and encodes a protein of 1980 amino acids with a predicted molecular weight of 220.0 kDa. The rat *Cacna1f* shares 88% and 95% identity with human (Genbank: NM_053701) and mouse (GenBank: NM_019582) sequences, respectively. The translated amino acids shares 91% and 97% with human (GenBank: NP_005174) and mouse (Genbank: NP_062528) sequences, respectively (Figure 4). Computational protein motif analysis showed four homologous domains of ion transport protein (Ion_trans), each containing six transmembrane alpha helices. The transmembrane segments were best conserved among species, and the most disparate regions were C-terminal and cytoplasmic loop between domains 2 and 3 [27].

### Mutation analysis

To identify the mutation responsible for CSNB rats, we amplified fragments encompassing *Nyx* or *Cacna1f* from cDNAs isolated from the retinas of control and mutant rats, which were identified by ERG analysis. Sequence analysis revealed a point mutation of C to T at position 2941, which changes codon 981 from arginine (CGA) to a stop codon (TGA). This R981Stop point mutation was predicted to lead to a version of protein shortened by a total of 999 amino acids, and missing the C-terminal and, in particular, part of the third and all of the fourth ion transport domains.

**Figure 5:**
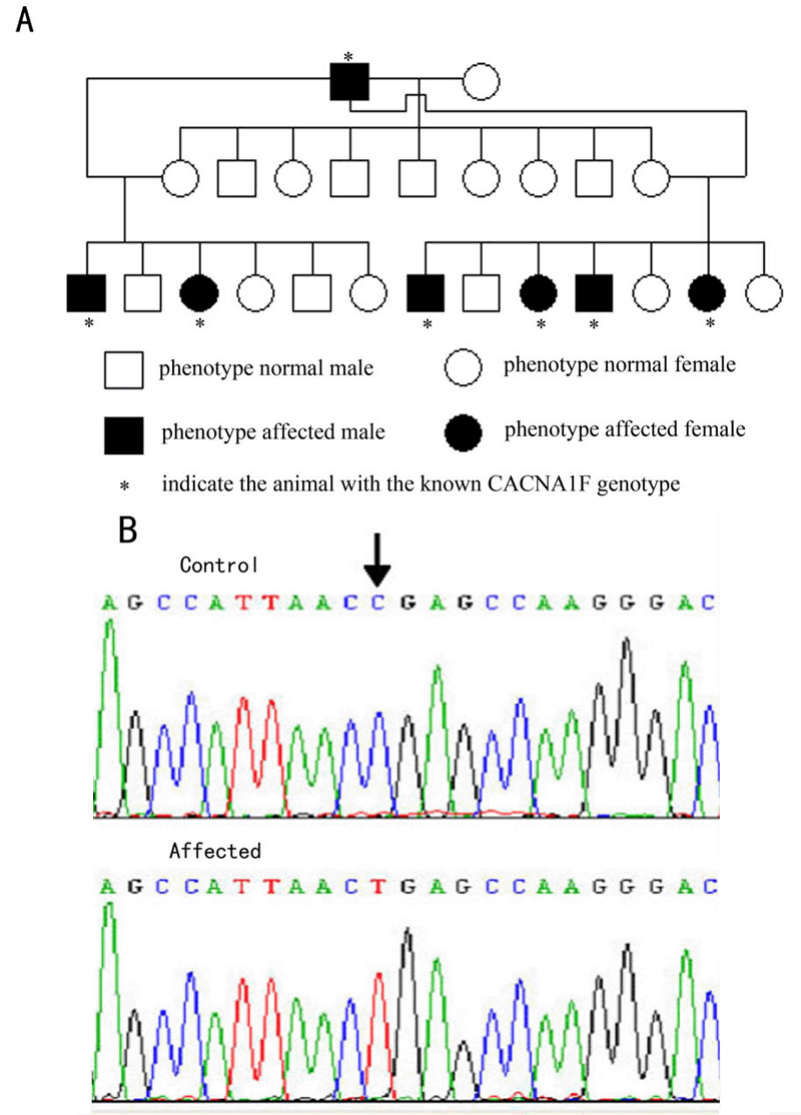
Mutation analysis of *Cacna1f* in CSNB rat. **A**: Pedigree was set up and it shows the complete cosegregation of the mutation with the phenotype. **B**: By mutation screening, a C>T mutation at position 2941 (p.R981Stop) was identified in affected animals. R represents Arginine.

To confirm that R981Stop was casually associated with the phenotype, we analyzed 24 rats obtained in our breeding pedigree (Figure 5). After ERGs were used to determine the phenotype, the mutant position was amplified from each animal's retinal cDNA. All seven affected rats were found to carry the c.2941C>T mutation in the retinal cDNAs, while no rat with a normal ERG carried this mutation.

### Histology and immunochemistry

**Figure 6:**
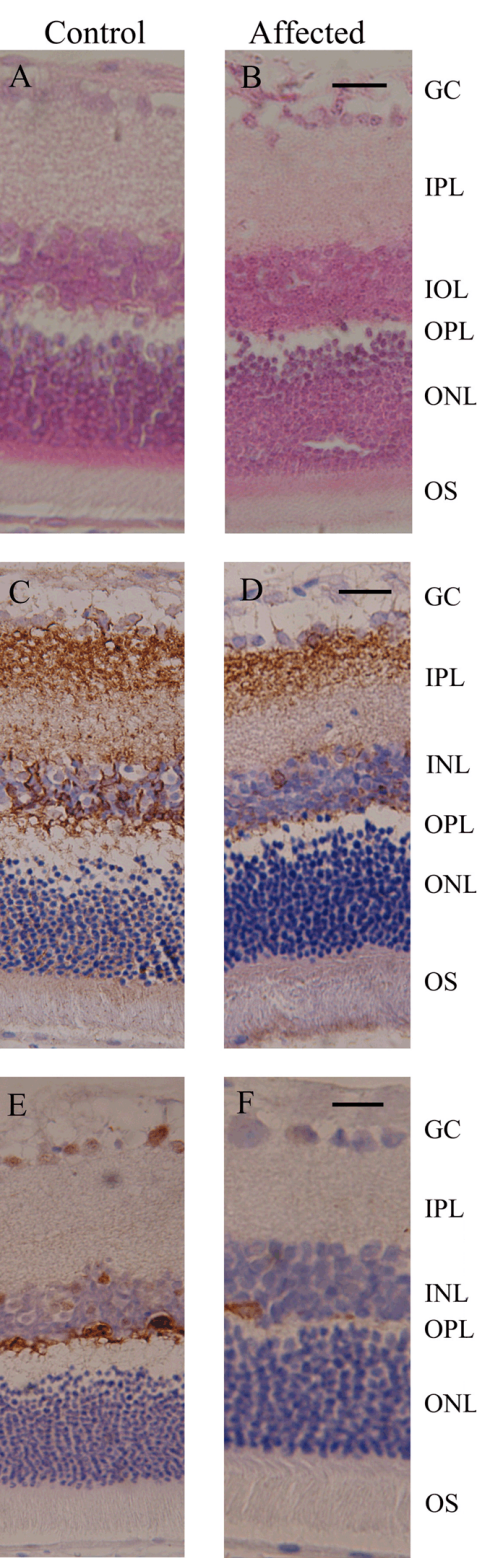
Histology and immunolabeling of retinal sections from control and affected rats. Retinal sections of control and affected rats were stained with hematoxylin and eosin (**A** and **B**). Bioplar and horizontal cells were identified with antibodies directed to PKCα (**C** and **D**) and calbidin (**E** and **F**). The following abbreviations are used: ganglion cells layer (GC), inner plexiform layer (IPL), inner nuclear layer (INL), outer plexiform layer (OPL), outer nuclear layer (ONL), and outer segment (OS). The scale bar represents 20 μm.

Histology showed that retinal structures were similar in both control and affected rats (Figure 6A,B). Labeling of control retinas with PKCα showed dendrites of bipolar cells terminated in the OPL (Figure 6C), in agreement with previous reports [28-31], whereas labeling with PKCα in affected retina was much reduced (Figure 6D). Labeling of control rat for horizontal cells with calbindin showed staining of bodies and processes in OPL (Figure 6E), in contrast, labeling for horizontal cells in affected retinas were rare (Figure 6F). In affected retinas, no extension to outer nuclear layer for dendrites of rod bipolar cells and horizontal cells were observed.

## Discussion

This study found that the rat mutant identified by ERG recordings [19] carried a mutation in the *Cacna1f* gene, while *Nyx* is normal. As a consequence, the rat model provides a new model for CSNB2, which also involves *Cacna1f* mutations. The protein encoded by *Cacna1f* is the α_1F_ subunit of voltage-gated L-type calcium channels, which appear to be expressed only in the retina [12]. Immunohistochemical analysis has localized this subunit to the ribbon active zones in rod photoreceptor terminals [32]. The visual signal generated by rod and cone photoreceptors in response to light is transmitted to second order neurons through glutamate released at ribbon synapses located in the rod and cone terminals [33]. Thus, a defect in α_1F_ would be expected to severely diminish post-receptoral transmission of the visual signal, which is clearly seen in all patients with CSNB2 and in the available animal models.

There are two mouse models for CSNB2. Mansergh et al. [30] described the phenotype of a knockout model (*Cacna1f*^−/−^), while Chang et al. [31] detailed a naturally-occurring mutant (*nob2*) identified, like the rat model under consideration, through fortuitous ERG studies. The rat phenotype appears to be an intermediate between these two mouse models. Under dark-adapted conditions, ERG b-waves and oscillatory potentials are essentially absent (Figure 1). This resembles the phenotype of *Cacna1f*^−/−^ mice [30] but not of the *nob2* mouse [31]. Under light-adapted conditions, affected rats generated clear cone ERGs of reduced amplitude (Figure 2). This phenotypic feature resembles more closely the results obtained in *nob2* mice [31] than of *Cacna1f*^−/−^ mice [30]. Immunochemistry showed that labeling for both rod bipolar cells and horizontal cells in affected retinas were reduced, especially for horizontal cells. This indicates that presence and activity of voltage-gated L-type calcium channels are essential for development of second-order neurons, such as bipolar and horizontal cells. Contrary to observations made in *Cacna1f* mutant mouse models, neither rod bipolar nor horizontal cells dendrites were observed to extend beyond the OPL in the rat. Given the range of phenotype seen in these mouse models and in human patients with Cacna1f mutations [14,15], the *Cacna1f* rat model will provide an additional animal model with which to understand the relationship between Cacna1f mutations and retinal phenotypes.
